# Automated workflows for strategy computation and data collection at synchrotron beamlines

**DOI:** 10.1107/S2059798326001920

**Published:** 2026-03-19

**Authors:** Rasmus H. Fogh, Peter Keller, Claus Flensburg, Clemens Vonrhein, Wlodek Paciorek, Leigh Carter, Gérard Bricogne

**Affiliations:** a Global Phasing Ltd 9 Journey Campus, Castle Park CambridgeCB3 0AX United Kingdom; Deutsches Elektronen-Synchrotron, Germany

**Keywords:** macromolecular X-ray crystallography, experimental strategy, automation

## Abstract

The *Global Phasing* workflow derives and executes experimental strategies for macromolecular crystallography, optimized in real time for the crystal symmetry and mounting orientation and the expected experimental resolution. By acquiring sweeps at multiple orientations, the workflow averages out the differences in Lorentz intensity enhancement and multiplicity to produce equally weighted merged reflections for processing. Values for various classes of parameters can be read from configuration files, submitted diffraction plans or user interaction, with optimal transmission settings derived from the beam flux density and the intended experimental resolution. The program can be executed in user-driven or unattended mode. Strategies are available for native or phasing experiments in either basic or advanced versions. The workflow has been shown to improve resolution, the number of unique reflections measured and average *I*/σ compared with a single-sweep experiment on equivalent crystals with the same total radiation dose. The workflow is a standalone program that could be interfaced to any beamline-control system, and is currently available through its integration with *MXCuBE* on a number of beamlines.

## Abstract 2

This paper presents and explains a workflow program for automatically deriving, optimizing and executing complex multi-sweep diffraction experiments for macromolecular crystallography at synchrotron beamlines.

## 1. Introduction

Crystallographic data-collection strategies aim at optimizing acquisition sweeps and parameters within the limits of the prevailing experimental conditions. The first acquisition-strategy programs for macromolecular crystallography came as parts of larger packages such as *MOSFLM* (Leslie, 1992 ▸) or *MADNES* (Messerschmidt & Pflugrath, 1987 ▸; Vicković *et al.*, 1994 ▸), with *STRATEGY* (Ravelli *et al.*, 1997 ▸) being the first standalone such program. The strategies determined by these programs were optimized for the experimental conditions used at the time: relatively noisy area detectors such as image plates or CCD detectors, single-axis goniostats with only the most minimal facilities for reorientation of the crystal, room-temperature acquisition with the concomitant high rates of radiation damage (at least for the first two programs mentioned) and experiment lengths limited by slow acquisition combined with a scarcity of beam time. The overriding consideration was to reach data completeness in the shortest possible time (before the crystal decayed) and the main tool available was to select the optimal starting rotation angle. The program *BEST* (Popov & Bourenkov, 2003 ▸) gives a detailed statistical study of the influence of the relevant parameters on different types of experiments, but the conclusion remained that shorter experiments were preferable. The work of Dauter (1999 ▸) gives a precise account of the minimum rotational range required for full completeness depending on crystal symmetry and orientation, this time from the starting point of selecting an appropriate orientation rather than making the best of the initial one. The introduction of the mini-κ goniostat (Brockhauser *et al.*, 2011 ▸) eliminated many of the practical problems associated with crystal reorientation; the discussion in the Brockhauser paper concentrates on its use for phasing experiments, on avoiding ‘blind zones’ (cusps) in the acquired data and on reducing spot overlaps.

The availability of experiments at cryogenic temperatures to reduce radiation damage, of fast, low-noise photon-counting detectors and of highly intense and stable beams, as well as the increasing adoption of multi-axis goniostats, have caused a fundamental change in the assumptions underlying previous acquisition-strategy approaches. We can now acquire complete data sets routinely on a single crystal in less than a minute, spread a given dose budget over many sweeps without suffering from detector noise and work easily with different crystal orientations. This makes it possible to also cater for hitherto unrealistic goals, such as spreading the acquired intensity more evenly across all the measured reflections and using low-dose-rate, high-multiplicity acquisition to mitigate the problems associated with radiation damage. The high acquisition speed and detector sensitivity has further meant that experimenters are ever less disposed to spend a long time setting up each experiment, at the same time as the average user has become less skilled in making optimal use of available instrumental capabilities.

Crystallography at room temperature remains an important area of research, but falls outside the scope of the present work. The need for humidity control and the much higher rate of radiation damage at higher temperature would require a different set of strategies, with a strong emphasis on combining data from multiple crystals.

The work presented here has been carried out with this new situation in mind, under the motto of ‘providing expertise without loss of automation’. The *Global Phasing* (*GPhL* for short) workflow program integrates with beamline-control systems to derive, as far as possible, the experimental plan and choices of parameter values that an experienced crystallo­grapher would propose, either as default values for the user to edit, or in full automation, and to then execute that plan. The *StratCal* strategy-generation program, which forms part of the package, specifically exploits the potential of multi-orientation experiments to deliver optimized strategies that could not have been achieved with a single sweep, and that would in practice have been too complex for users to set up on their own.

## 2. The *GPhL* workflow process

**Figure 1:**
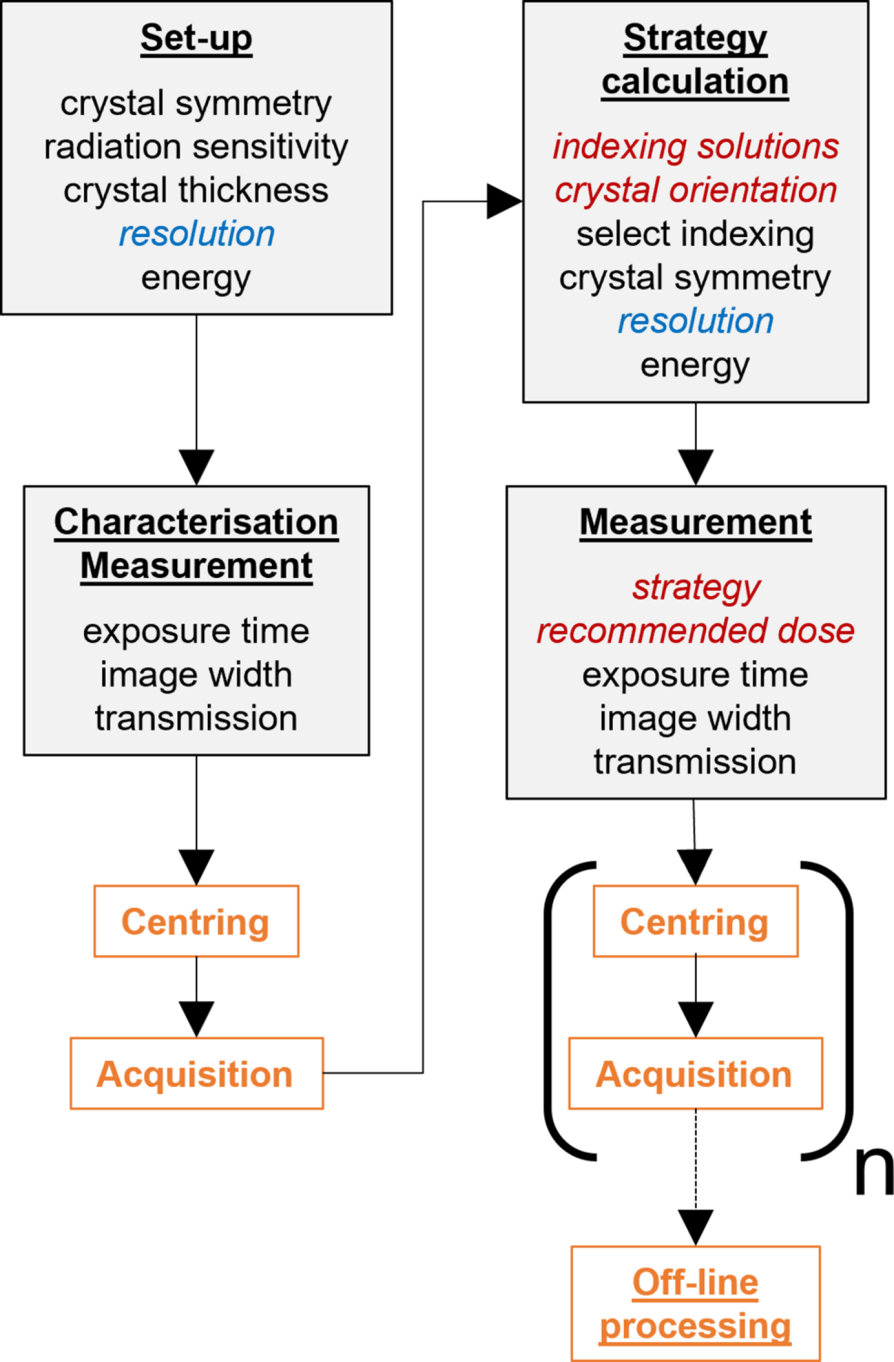
Sequence of the *GPhL* workflow. Light grey boxes represent workflow steps, showing input parameters; orange boxes are actions outside the workflow software. Input parameters in black have default values from configuration data and can be reset manually from the user interface or from the diffraction plan in automation. Parameters in red italics are calculated from earlier workflow steps. The resolution is the only parameter that cannot be reliably set by configuration and must be set for each sample. In the implementation at MASSIF-1 (Bowler *et al.*, 2016 ▸), all steps up to the characterization and the calculation of the indexing solutions and crystal orientation are performed before the *GPhL* workflow is started, and the information is transferred to the workflow, which starts by selecting the indexing solution.

The actions of the *GPhL* workflow can be seen in Fig. 1 ▸. The strategy calculation relies on the crystal orientation matrix and point-group symmetry (more exactly, on its arithmetic crystal class; see below) as input. The workflow uses a characterization strategy of multiple small wedges spread over an ω range of 180° (typically five wedges of 12 images, 0.1° per image) to provide a more robust characterization than the more common approach of using two wedges only.

## 3. Input parameters

### 3.1. Resolution

The target resolution is a key input parameter, both for setting the detector distance and for calculating the optimum strategy and transmission. In the current workflow version this is the only mandatory input parameter that must be set separately for each sample, either manually or from the diffraction plan. Future program versions should derive a default value for the target resolution from the diffraction data, either through direct analysis of the characterization images or through external programs such as *DOZOR* (Zander *et al.*, 2015 ▸; Svensson *et al.*, 2015 ▸).

### 3.2. Indexing and symmetry

In order to generate the optimal strategy, *StratCal* needs the point group of the crystal and the orientation of all of its symmetry axes as input. In some cases (specifically, for point group 32) additional information is needed about the lattice organization in order to locate the symmetry axes in the unit cell. This additional information is captured by specifying symmetry in terms of an arithmetic crystal class (Wilson, 2006 ▸). The indexing solution is determined by running the first steps of *XDS* and selecting an indexing solution from the resulting table (either automatically or manually), taking into account the expected symmetry information that can optionally be specified as part of the sample-shipment data at the set-up stage. When an expected symmetry is not known exactly, or when there is no matching indexing solution, there are a number of complications. The input information may be ambiguous, or pseudo-symmetry (translational or rotational) in the crystal may be known to lead to incorrect Bravais lattice identification. Where there is no prior symmetry information that matches the indexing results, the Bravais lattice does not provide sufficient information to determine the point group, nor can it be determined unambiguously. For instance, an indexing solution with three orthogonal axes, two of which have the same length, could correspond to point groups 4 or 422, 222 (with two different settings) or 2 (with three different orientations of the twofold axis). To deal with this problem, the *GPhL* workflow specifies symmetry as a list of possible arithmetic crystal classes compatible with the axes of the selected indexing solution, that each determines a combination of point group, centring and axis orientations. In interactive mode the user can select relevant combinations of crystal classes. *StratCal* can then use this list to derive a strategy that will give good results for all symmetries in the list. In the current version, *StratCal* assumes the lowest symmetry above monoclinic (*e.g.* 3 rather than 6, 32 or 622 in the case of a hexagonal lattice), but in some cases, such as the (pseudo?) tetragonal case given above, it will be possible for future versions to generate a single strategy that will be close to optimal for either of several alternative symmetries.

### 3.3. Transmission and radiation damage

It is radiation damage that determines the maximum number of photons that can be passed through a crystal before useful signal decays below a given threshold, so an acquisition strategy needs to determine the maximum tolerable dose (according to some acceptability criterion) and make the best possible use of it. The rate of radiation damage depends on the absorption of the crystal, which is determined by the wavelength and the crystal chemical composition. The workflow accounts for this using a single input parameter at the set-up stage, the radiation sensitivity relative to a protein crystal of standard composition.

The workflow calculates a recommended dose budget and a matching default transmission setting that is optimized for the target resolution. The transmission can be overridden either interactively or from the diffraction plan. The critical parameter in our criterion is the fraction of the initial intensity at the chosen target resolution that must remain in the last images of the experiment. The cutoff value can be configured; by default, it is set, heuristically, to 25%.

For data acquisition at 100 K the dose-dependent decrease in intensity is given by
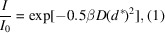
where *D* is the accumulated dose, d* is the reciprocal resolution of the relevant reflection and β is an empirical constant with a value of 1.0 Å^2^ MGy^−1^ (Leal *et al.*, 2011 ▸). The experimental temperature (like other instrument parameters) is entirely under user control, but the workflow is designed for low-temperature experiments and does not calculate recommended dose budgets or transmission values for room-temperature acquisition. For room-temperature experiments users would have to disregard the proposed values and determine the appropriate transmission independently.

If *F*(*v*, *t*) is the instantaneous flux density at voxel *v* and time *t*, then
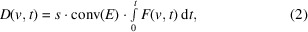
where *s* is the relative radiation sensitivity of the crystal, *E* is the beam energy and conv(*E*) converts between the flux (in photons s^−1^) and dose (in MGy) for an ‘average crystal’ of sensitivity 1.0. The relative intensity for the entire sample is found by integrating over all voxels *v* in the crystal volume *V*, as
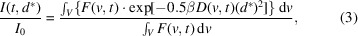
which can be written in terms of the relative flux density per voxel and time unit, *f*(*v*, *t*), as
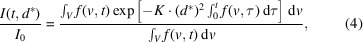
where
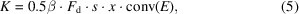
where *x* is the fractional transmission and *F*_d_ is the flux density at 100% transmission.

**Figure 2:**
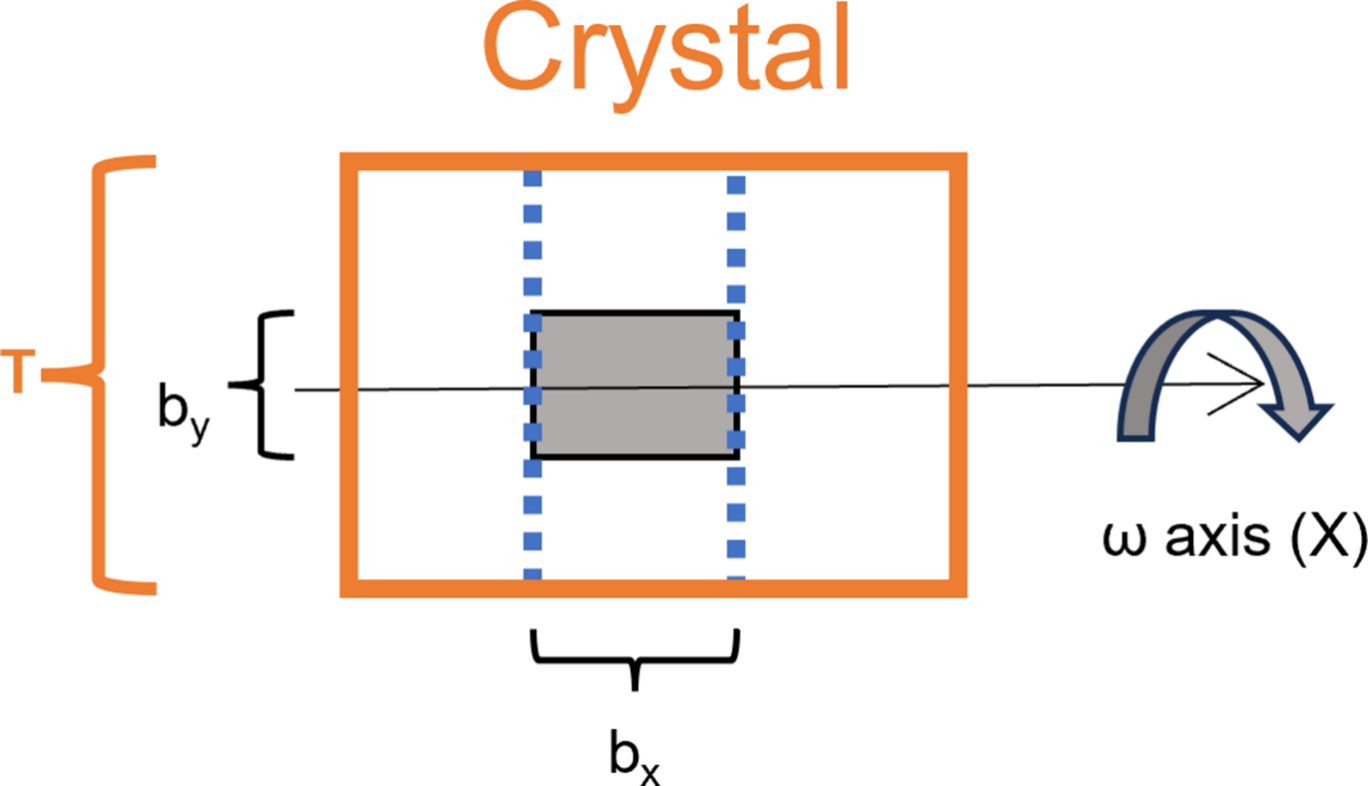
Crystal of thickness *T* rotating around ω. The beam is in the *Z* direction (out of the paper) with size *b*_*x*_*b*_*y*_. The slice of the crystal between the two blue dashed lines will be irradiated as the crystal rotates.

If the beam profile, the crystal shape and orientation and the movement of the crystal throughout the experiment are known (including centring points for each sweep), the *RADDOSE*-3*D* program (Zeldin *et al.*, 2013 ▸) can be used to calculate the relative intensity decay factor exactly. While this could be performed after the fact, it is unfortunately impractical to gather such detailed information before the experiment starts, which is when one needs to calculate the required transmission setting. For practical reasons, we would want to approximate the problem by the uniform application of a single ‘effective’ flux density *F*_d_. This would simplify the formula to 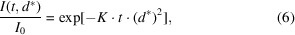
which can be used easily to calculate transmission values for any combination of parameters. The question is whether we can find a useful estimate for the ‘effective’ flux density. If we have no information about the crystal size, the best we can do is to use the average flux density across the beam, as if we were using a top-hat beam that fully bathed the crystal. In terms of the parameters given in Fig. 2 ▸, this gives a formula for the effective flux density of
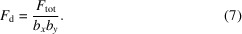
For beams that are not much smaller than the crystal and that have relatively uniform flux density, this has proved to give reasonable results. For beams that are highly peaked or much narrower than the crystal, the resulting values can be quite unrealistic. Doing better requires taking account of the crystal size. To circumvent the need to know the precise size and shape of the crystal, we will optimize the transmission based on the narrowest part of the crystal: the point in a 360° sweep where the irradiated material is the least and where the measured signal is at a minimum. This requires only one parameter to be input, the ‘crystal thickness’ *T*, and corresponds to treating the crystal as a roughly cylindrical rod of this thickness oriented along the rotation axis. The resulting formula can be explained by looking at Fig. 2 ▸. It is clear that the beam photons will all traverse the slice of the crystal of width *b*_*x*_ and diameter *T*. We will therefore approximate the actual beam by a uniform beam with flux density
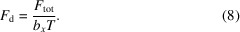
It should be noted that the correction only applies in cases where the effective beam is narrower than the smallest dimension of the crystal. For, for example, plate-shaped crystals where the ‘width-to-thickness ratio’ is large, the transmission will be optimized to the part of the crystal that is continuously irradiated and where the signal is smallest and decays most rapidly, and the larger signal that arises when the wider part of the crystal is rotated into beam will be taken as a bonus. To deal with variations in beam shape, we will treat narrow Gaussian beams as equivalent to top-hat beams with the same total flux and a width equal to the full-width half-maximum of the Gaussian.

**Figure 3:**
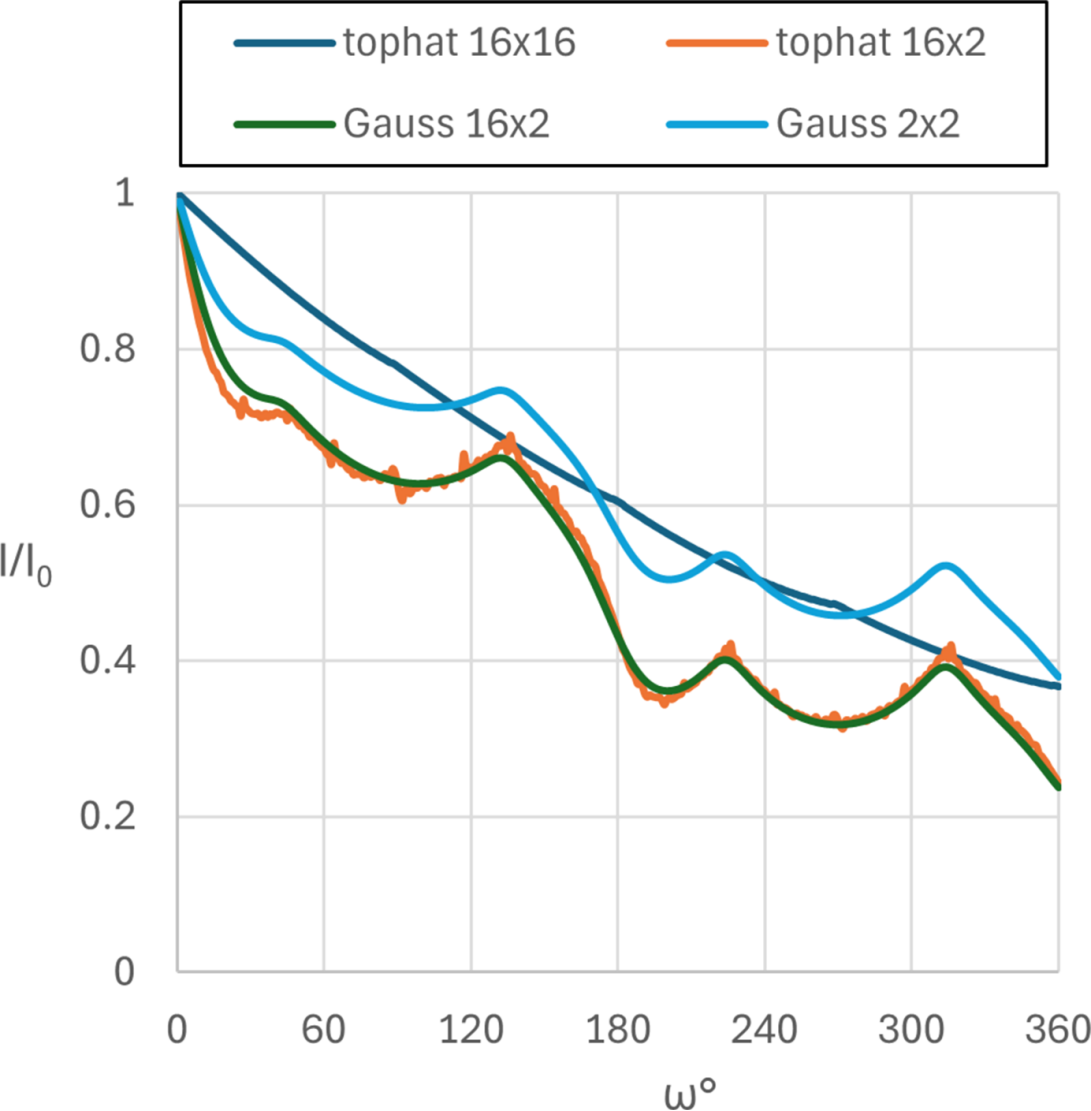
*RADDOSE*-3*D* simulations of relative intensity versus rotation angle for four different beam shapes. All simulations were performed for a 12 × 12 × 12 µm cube-shaped crystal rotating around one of its axes. The total flux was 4 × 10^9^ photons s^−1^ for all simulations except ‘Gauss 2×2’ where it was 5 × 10^8^ to compensate for the narrower width of the beam in the *X* direction. The features 90° apart on some of the curves arise from the corners of the cubic crystal moving in and out of the beam.

Fig. 3 ▸ shows *RADDOSE*-3*D* simulations for beams that should be equivalent according to this approximation formula. The top-hat 16 × 16 reference beam gives a smooth decay curve, as it corresponds to a fully bathed crystal. The other curves come from beams that are a factor of eight narrower orthogonal to the rotation axis. They are characterized by an initial rapid decay, as the centre of the crystal is damaged, followed by a stair-shaped curve with a step every 180°. As the crystal rotates, fresh crystal will continuously be brought into the beam at the crystal periphery; this keeps the relative intensity more or less constant until the rotation reaches 180°, where the unirradiated crystal runs out.

The simulations show that the approximate formula given here can be relied on to give reasonably good estimates of the effect of transmission settings. The two beams that have the same width along the rotation axis as the reference beam give identical results, showing that narrow Gaussians are well approximated by a top-hat beam of the same width. The curves are lower than reference, but match at the end of each ‘step’. The curve for a 2 × 2 Gaussian beam, which is rather more realistic than the 16 × 2 Gaussians discussed above, fits the reference curve much better, presumably because the errors in assuming a uniform flux density throughout the irradiated slice are partially compensated by the errors in ignoring the tails of the Gaussian in the *X* direction.

So far we have not considered the effect of combining sweeps with multiple orientations and possibly multiple centring points; we would simply use the approximation formula as if the experiment was a single long sweep. In the case of a pseudo-helical experiment, it would not be hard to calculate transmission on the basis that each sweep was applied to a fresh section of the crystal. The available information would not justify an attempt to refine the treatment further. For more precise results (if desired) it would be necessary to measure the crystal and beam properly and apply a full *RADDOSE*-3*D* simulation.

## 4. Experimental strategies

Acquisition strategies cannot be optimized against a single criterion, but must balance a number of factors. Most obviously there is completeness, multiplicity of measurement and uniformity of its distribution, goniostat shadows, effective scaling calculations, radiation damage and overall experiment time. For phasing experiments, there is the overriding consideration of the clean subtraction of measurement pairs from which anomalous differences are derived, in order to ensure that systematic errors from radiation damage that affect each member cancel out of their difference. The effect of acquisition strategies can best be viewed by plotting all reflections in reciprocal space and marking them according to multiplicity or measured signal-to-noise (S/N) enhancement. The orientation of the φ axis is fixed to the crystal at mounting time, which provides the location of the unmeasured cusps for χ = 0°. A multi-axis goniostat allows one to change the orientation of the rotation axis by up to χ_max_ in any direction away from the starting position. The value of χ_max_is 48° for a standard mini-κ goniostat, and in practice is set to about 45° for a Smargon goniostat for reasons of collision avoidance. This translates to around 33% of the possible crystal orientations being available for any given crystal mounting.

**Figure 4:**
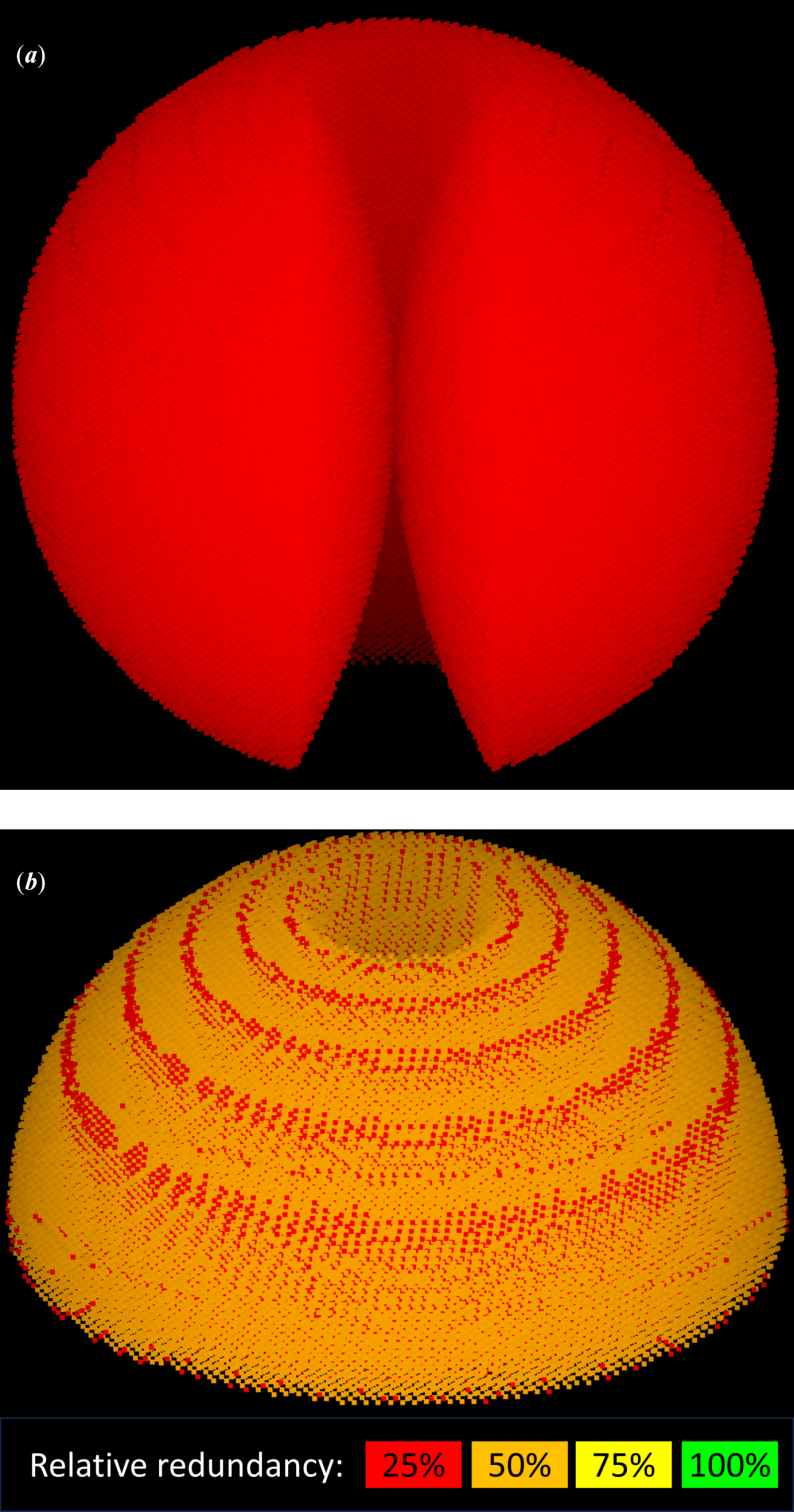
Raw reflections (*a*) and merged reflections (*b*) as measured by a 180° sweep for a triclinic crystal. The experiment was simulated with the *GPhL* program *SimCal*, using ray tracing, for a Dectris PILATUS 6M detector (Dectris, 2025 ▸), with a 1 Å wavelength and a limit resolution of 1.93 Å, to give a maximum reflection angle 2θ of 15°. (*a*) shows all raw measured reflections. A trumpet-shaped cusp of reflections around the rotation axis cannot be measured regardless of the length of the sweep. The two cusps are connected by a ditch of unmeasured reflections, which is counterbalanced by a ridge of symmetry-equivalent reflections that are measured twice (not visible in the figure), giving 100% non-anomalous completeness for all reflections outside the cusp. (*b*) shows the merged reflections for one hemisphere, colour-coded by redundancy relative to a 360° sweep. One of the cusps is clearly visible; the red rings of lower multiplicity arise from module gaps on the detector.

The basic building block we use for strategies is based on the 180° sweep (see Fig. 4 ▸), which gives 100% (non-anomalous) completeness except for reflections in the two cusps. The effect of crystal symmetry will be equivalent to combining the effect of a single sweep and all of its symmetry images.

Some of the relevant factors are relatively easy to account for. High completeness requires ensuring that the cusp of unmeasured reflections is compensated, preferably by symmetry-equivalent reflections measured in the same sweep. High multiplicity requires longer measurements at lower dose rates, as does the avoidance (or at least reduction) of overloaded reflections for the few, high-intensity reflections where these may still be encountered. Radiation damage can be minimized by appropriate setting of the transmission and, again, by measuring many images at low dose rates so that damaged images can be removed during processing without compromising the overall completeness. Experiment throughput, on the other hand, requires shortening the experiment, reducing the total length of the sweeps and the number of recentring operations required to support multiple orientations. The time needed for recentring can vary significantly depending on goniostat technology (mini-κ versus Smargon), recentring procedures (manual, X-ray or optical) the reproducibility of calculated centring positions and the precision required by beam and crystal dimensions. In one case, a 24-hour protein crystallography session at EMBL-HH-P14 under production conditions, it was found that the time required for one manual recentring was about 45 s, similar to the time it took to acquire a 180° sweep. Other considerations require a more detailed treatment.

### 4.1. Intensity averaging

Data processing and subsequent calculations are more robust if all reflections are acquired with equal weight. The weight of a merged reflection depends first on its resolution, but also on the number of independent reflections that went into the merging, *i.e.* on the multiplicity. It also depends on the sum of the Lorentz intensity enhancement factors for the reflections. The product of the relative multiplicity and the average Lorentz enhancement will be termed the S/N enhancement factor. The workflow uses multiple sweeps at selected orientations so as to average out both the number of times a reflection is measured and the intensity enhancement it undergoes.

**Figure 5:**
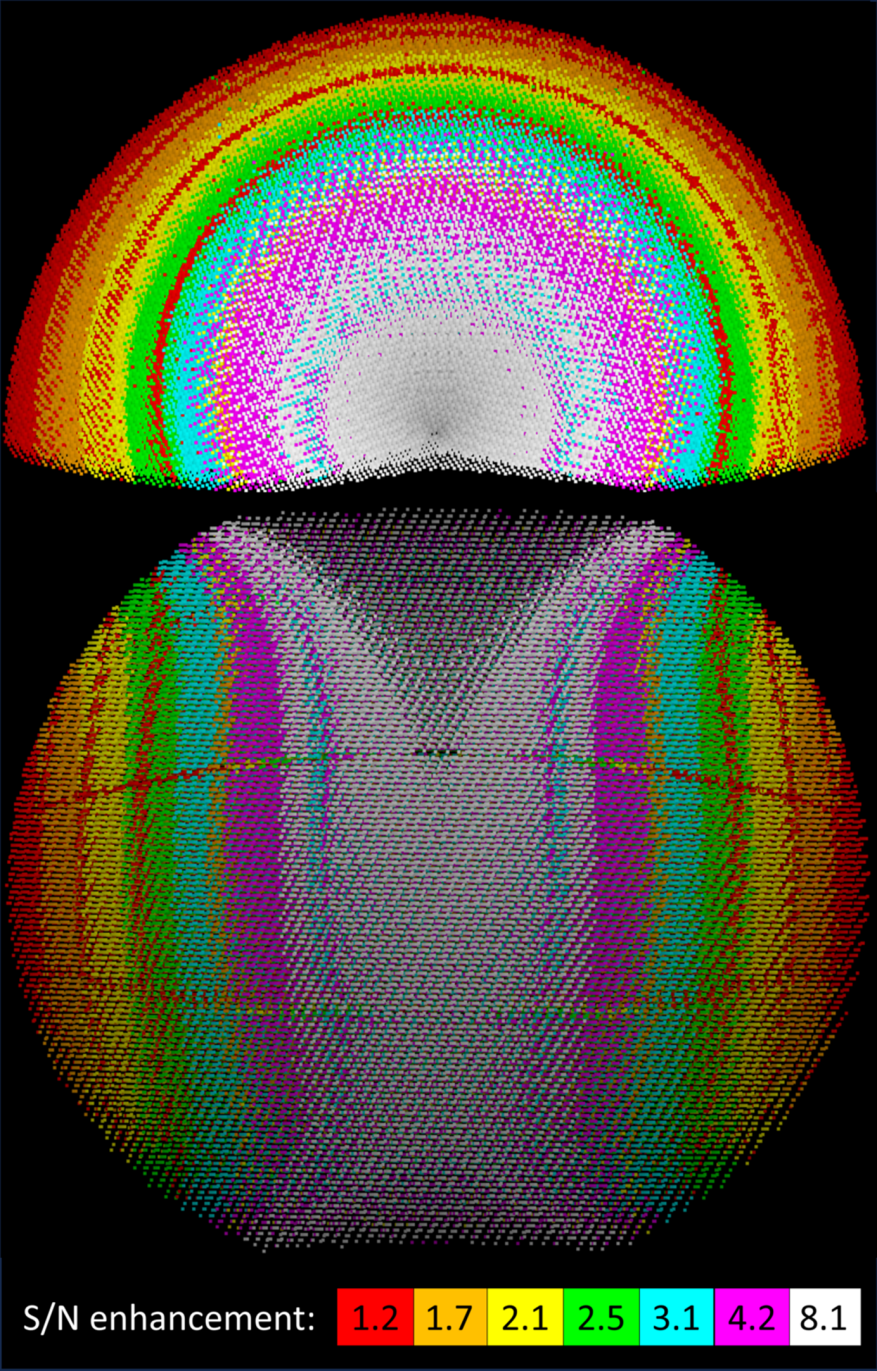
Relative S/N enhancement due to the Lorentz factor and multiplicity of measured reflections for a 180° sweep on a triclinic crystal. Simulation parameters are as for Fig. 4 ▸, except that the limit resolution is 1.0 Å, giving a maximum reflection angle 2θ of 30°. The half-sphere of unique reflections is seen from above the cusp of missing reflections (top) and from the middle of the sphere of reflections (bottom). Reflections are grouped into equi-populated, colour-coded bins, with median enhancement factors (Lorentz times multiplicity) for each bin as shown in the figure. The most intense white point has an enhancement factor of 105. The grid of lower intensity points on the bottom and the red circles visible on the top figure reflect the image of the detector-module gaps, where the reflection redundancy is 1 instead of 2. Enhancement factors increase for a given reflection as one moves closer to the rotation axis until the cusp is reached.

The intensity enhancement of a reflection depends on how long it takes for the reflection to move through the reflection condition, with slow-moving reflections in effect being measured for longer. This effect, known as the Lorentz intensity enhancement (*L*), in turn depends on the resolution of the reflection and its location on the Ewald sphere. For a rotation axis orthogonal to the beam, the effect is given by Holton & Frankel (2010 ▸) as
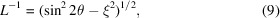
where ξ is the projection of **R**, the reciprocal-space reflection vector normalized to λ = 1, onto the rotation axis. From the diffraction condition we have
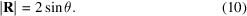
Denoting by 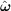 a unit vector along the direction of the rotation axis, this gives
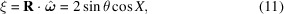
where *X* is the angle between the reciprocal-space reflection vector and the rotation axis. Combining equations (9) and (11) gives
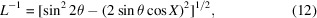
leading to
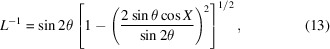
which finally gives
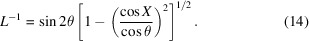
While the resolution dependence of the intensity enhancement is a given, it is possible to average the effective S/N enhancement of a merged resolution by measuring it in a variety of orientations with different values of *X*, close to or far from the rotation axis and the cusp. In addition to the effect of the Lorentz factor, this will also average out the reflections that are missing due to cusps or detector-module gaps. The overall S/N enhancement distribution can be seen in Fig. 5 ▸.

### 4.2. Goniostat shadowing

**Figure 6:**
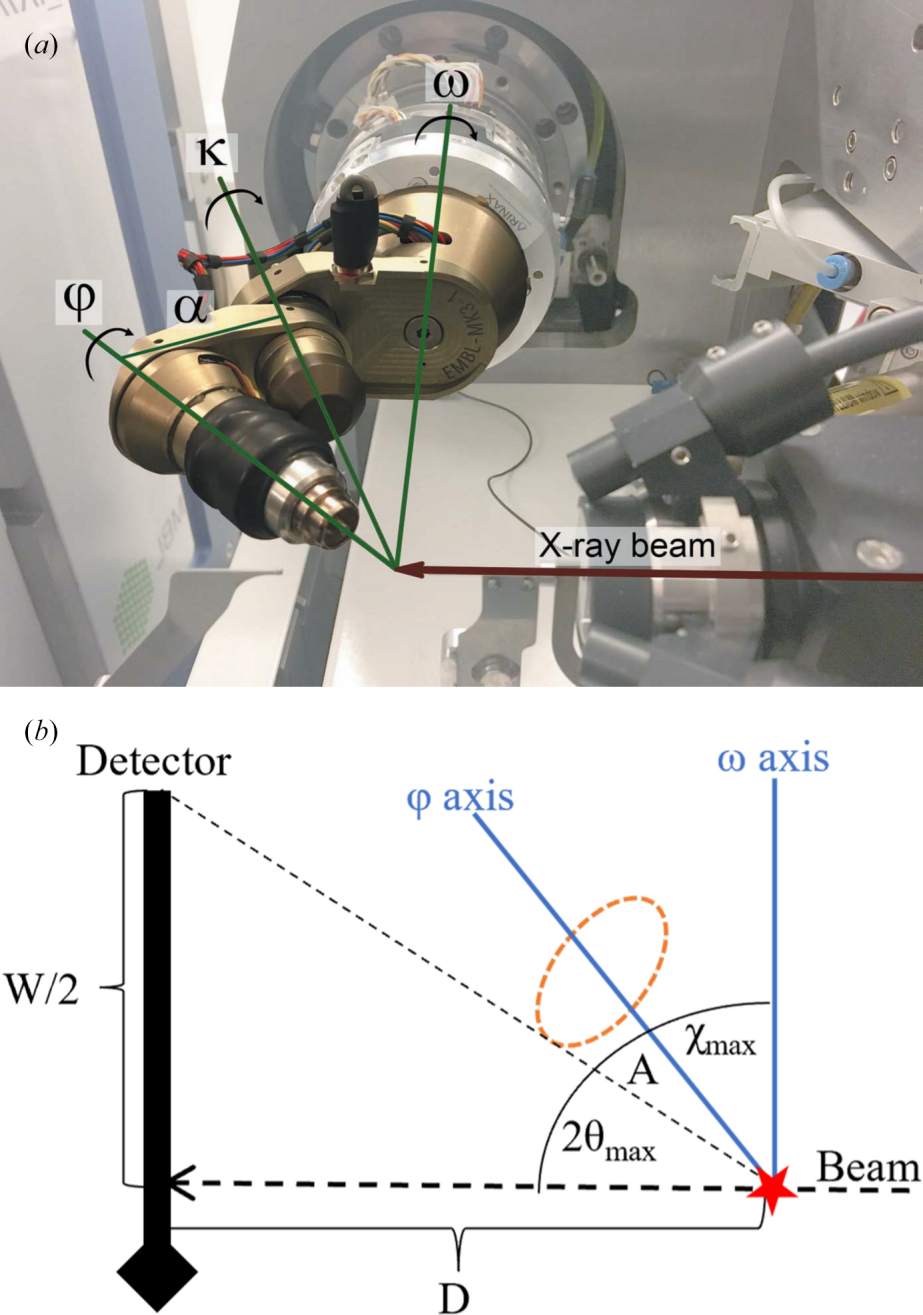
(*a*) Arinax mini-κ goniostat at maximum extension (κ = 180°), as used at the ESRF ID30B beamline (White *et al.*, 2018 ▸). The detector is at the left of the picture. χ is the angle between the ω and φ axes. It can be seen that for short detector distances and high χ angles the φ-axis mounting may block some of the reflections passing from the diffracting crystal to the detector as the sample is rotated. (*b*) Goniostat positioned with the φ axis in the plane of the beam and the ω axis at its closest approach to the detector. The detector distance *D* is set to the shortest value that avoids creating goniostat shadows. The red star at the lower right shows the crystal. The orange oval describes the outer envelope of the φ-axis mounting. *A* is the angle between the φ axis and the outer envelope, which is 19.84° for a mini-κ goniostat. Smargon goniostats have a different construction, but the main shadow from the φ-axis mounting is a cone with a similar aperture angle as for the mini-κ. 2θ_max_ is the highest angular deviation for a reflection that does not fall outside the detector, corresponding to the targeted resolution limit, and χ_max_ is the highest value of χ that permits a 360° sweep without causing goniostat shadows.

At high χ values and short detector distances the body of the goniostat may move between the crystal and the detector, giving rise to ω-dependent shadows on the detector, as shown in Figs. 6 ▸ and 7 ▸. These shadows reduce completeness and can have a deleterious effect on processing, as unless these shadows are masked, some data-processing packages may produce spurious integrated intensities of close to zero for shadowed reflections as if they had reached the detector unimpeded. Given a solid model of the goniostat and a precise calibration of its axes, the *Global Phasing* processing program *autoPROC* is capable of generating per-image exclusion lists for the unobserved reflections, which can be read by *XDS* (as ‘FILTER files’) since the November 11 2013 version (Kabsch, 2010 ▸; https://xds.mr.mpg.de/html_doc/Release_Notes.html). Nevertheless, it is preferable for acquisition strategies to minimize goniostat shadowing.

**Figure 8:**
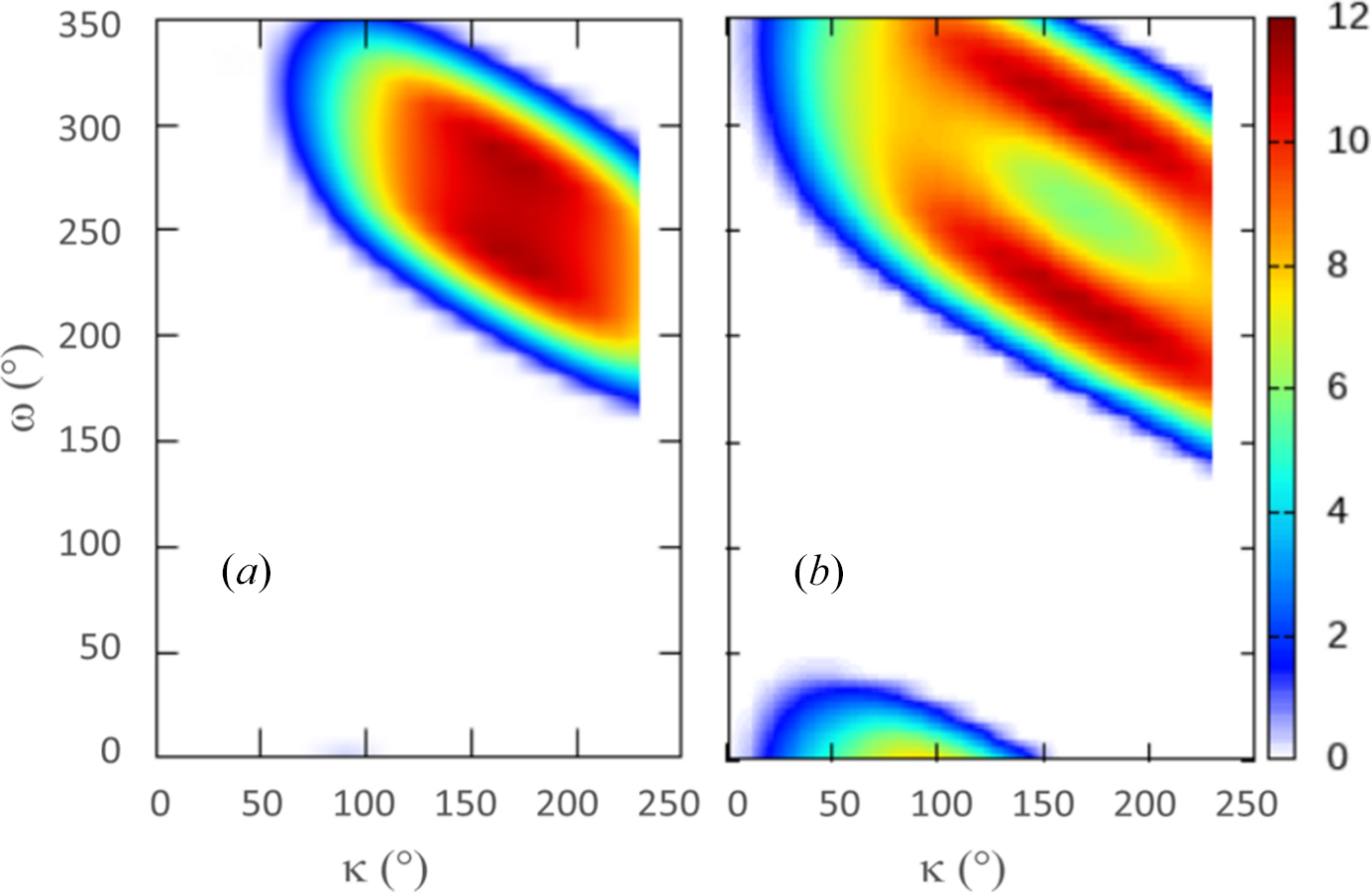
Percentage of shadowing on the detector for different ω and κ. (*a*) PILATUS3 6M detector at *D* = 200 mm, equivalent to an EIGER 16M detector at 150 mm. (*b*) PILATUS3 6M detector at *D* = 100 mm (excluding minor κ-independent shadows from the κ bracket). The shadow-free ω range is about 225° in (*a*) and 185° in (*b*) for a range of κ values from 90° to 240°. The PILATUS3 6M detector is the largest currently available from Dectris, and the detector distance of 100 mm in (*b*) is too short for realistic operation; the shortest detector distances observed have been in the range 135–150 mm.

Fig. 6 ▸(*b*) shows the goniostat set to the highest resolution that allows a 360° sweep without goniostat shadows. Given that the detector distance is set so that the target resolution *d*_min_ is just within the limit of the detector, we have
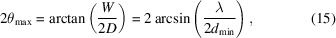
where the parameters are defined in the legend to Fig. 6 ▸(*b*). *d*_min_ corresponds to the actual resolution of the reflection. Representative examples of the highest achievable values of 2θ_max_ are shown in Table 1 ▸. Except for the particularly large PILATUS3 6M detector one can acquire a shadow-free 360° sweep out to χ = 20–30°, and one can reorient the crystal by θ_max_, as required in the worst case to move the cusp away from a symmetry axis, while causing only a minimal goniostat shadow. For larger χ angles, the φ axis mounting will come between the crystal and the detector for some ω values. It is clear from Fig. 8 ▸ that a 192° sweep at a suitably selected ω starting angle can be carried out with at most minimal goniostat shadowing at even the highest achievable resolution, for a centred detector.

### 4.3. Scaling calculations

The total diffraction intensity of the crystal will vary over time depending on changes (decay) in beam intensity, the amount of crystal material in the beam at any given time and the intensity decay caused by radiation damage. It is therefore necessary to scale different images against each other as part of data processing so that the intensities of reflections from the images can be compared. This requires that the same reflections be measured on different images. During a sweep, reflections *h*, *k*, *l* and −*h*, −*k*, −*l* (which have the same intensity in the absence of anomalous diffraction) will be measured in images down to 2arcsin[λ/(2*d*_min_)] apart, which allows relative scaling of images that are acquired reasonably close to each other. Sweeps with the crystal at different orientations will have images oriented differently in reciprocal space, so that each image from one set should have a few reflections in common with any image from another set. Yet, in order to achieve more robust and reliable scaling it is preferable to have a strong overlap between images that are far apart within a single sweep, so as to anchor the relative scaling constants. To this end, the native strategies are organized in such a way that the first sweep is of length 360° at a relatively low χ, which gives robust scaling across the entire sweep. For sweeps at higher χ, where shadowing does not allow the use of 360° sweeps, the strategies use a sweep length of slightly above 180°, so that the end of the sweep can be scaled precisely to the beginning. A further detail is that the *XDS* processing program calculates scaling in batches of about 5°. For fast parallel processing it is preferable that the sweep length corresponds to an integer number of batches, and that the number of images in a batch is an integer multiple of the number of processors used. The relevant values can be configured to a specific beamline setup; by default, *StratCal* is set to a batch size of 4.8° and sweep widths of 192° (or 360°).

### 4.4. Basic native strategies

The basic strategies aim to be fast, while providing 100% completeness without cusps and at least twofold redundancy of acquisition, so that the loss of some images to radiation damage or bad crystal centring will not risk reducing the completeness. This can be performed by acquiring a single 360° sweep at χ = 0°, except for reorienting the crystal far enough away from unique symmetry elements to ensure that all reflections in the cusp are measured in a symmetry-equivalent position elsewhere. For the special case of *P*1 symmetry, at least two sweeps are necessary in order to measure all reflections. Avoiding reorientation where possible reduces the number of (time-consuming) recentring operations, and acquiring at low values of χ effectively avoids the problem of goniostat shadowing. The closest equivalent approach would be the practice of acquiring two sweeps at a fixed relative angle of, for example, 30°, as used, for example, at the Diamond Light Source I04 beamline (Diamond Light Source, 2024 ▸). This alternative avoids the need for robust characterization and determination of the crystal orientation matrix. Unlike the *GPhL* approach it is not, however, guaranteed to avoid cusps of missing reflections at high resolution and always requires recentring once.

### 4.5. Advanced native strategies

**Figure 9:**
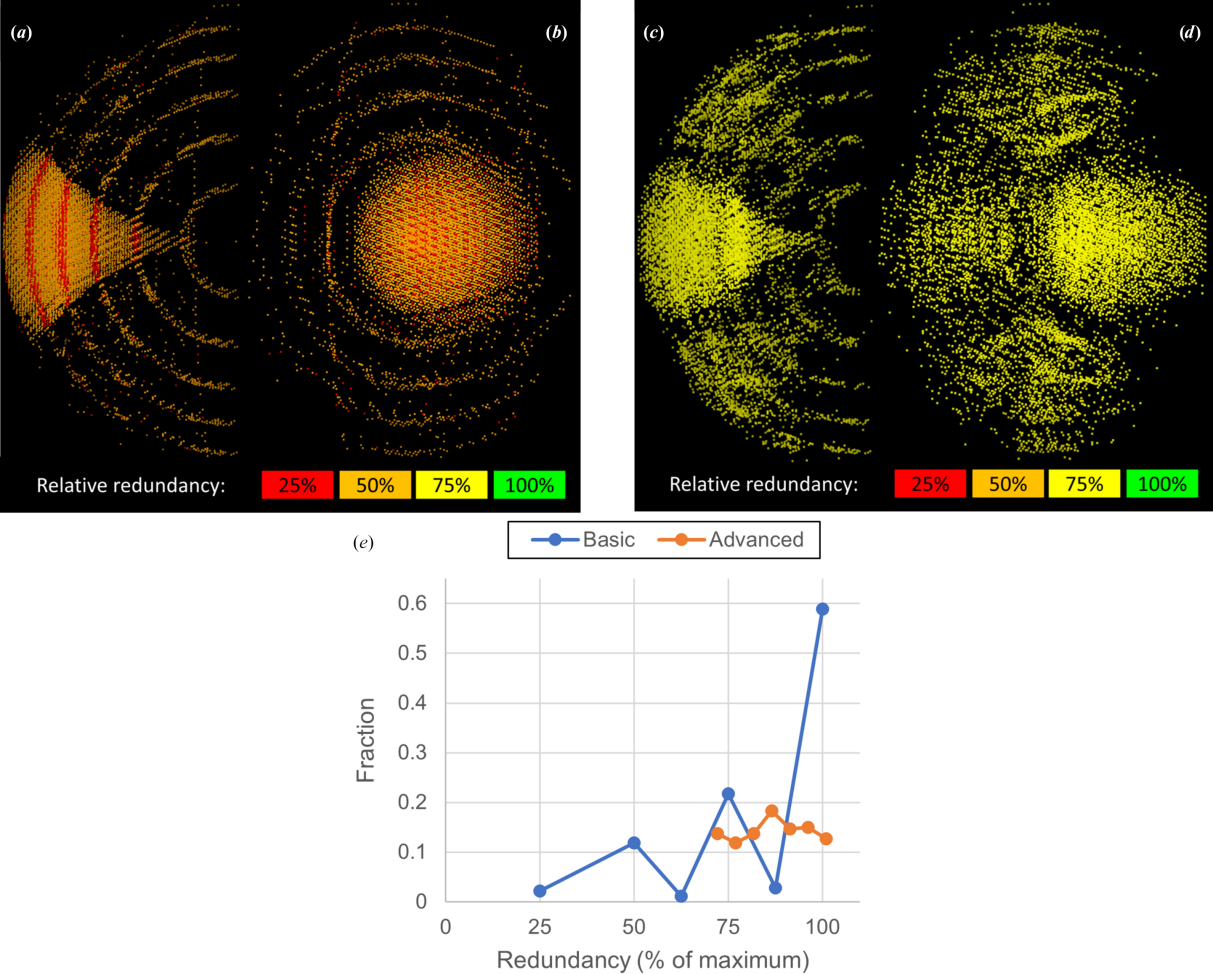
Strategies and redundancies for a monoclinic crystal. Simulation parameters are as for Fig. 4 ▸, except that the limit resolution is set to 1.22 Å, giving a maximum reflection angle 2θ of 24.2°. (*a*)–(*d*) Orthogonal views of the 14% lowest redundancy reflections for a basic strategy (*a*, *b*) and an advanced strategy (*c*, *d*). The strategies are: basic, 360° in one sweep at κ = 0°; advanced, 936° total with one 360° sweep at κ = 0° and three sweeps of 192° each with κ = 180°. Going from a basic to an advanced strategy increases the lowest redundancy from 25% to∼75%, due to averaging over multiple orientations. Note the concentric rings arising from reflections lost in the module gaps, and the trumpet-shaped images of the cusps. (*e*) Fraction of reflections per redundancy bin for a basic and advanced strategy simulation. The redundancy distribution is much more uniform for the advanced strategy. As the advanced strategy length is not an integer multiple of 180° the maximum redundancy has been set to 936°/360° times the maximum redundancy (8) for the basic strategy. This explains the value of slightly above 100% for the highest redundancy bin.

The aim of advanced native strategies is to combine multiple sweeps and their symmetry images so as to generate a complete coverage of reciprocal space with a uniform distribution of S/N enhancement. This amounts to combining a number of distributions such as described in Fig. 5 ▸ into a uniform spherical distribution. Considering the highly irregular shape of the initial distribution, not least the sharp discontinuities close to the cusps, this problem clearly does not have a unique, clear answer. The gaps between detector modules also reduce the redundancy of the measured reflections. The percentage of the detector area that is inactive due to module gaps can be as high as 7–9% for large EIGER or PILATUS detectors. Since integration requires measuring typically 75% of the reflection intensity, the actual percentage of excluded reflections is higher, calculated as ∼10% by simulation for a PILATUS 6M detector. The effect on overall completeness is much smaller. With correct positioning of the beam centre, even a single-sweep experiment can achieve a redundancy of at least one for all reflections outside the cusp, and crystal symmetry will further improve the situation. Fig. 9 ▸ shows the distribution of redundancy for a single-sweep strategy versus a multi-sweep strategy for a monoclinic crystal. For a single sweep the redundancy is dominated by the effect of detector-module gaps (reducing the redundancy from 8 to 6 for a 360° sweep) and of the cusps of unmeasurable reflections (reducing the redundancy from 8 to 4). The four-sweep advanced strategy averages out the extremes of redundancy, resulting in a range of redundancy values from 6 to 8, with a median of 7, when rescaled to the same maximum redundancy.

**Figure 10:**
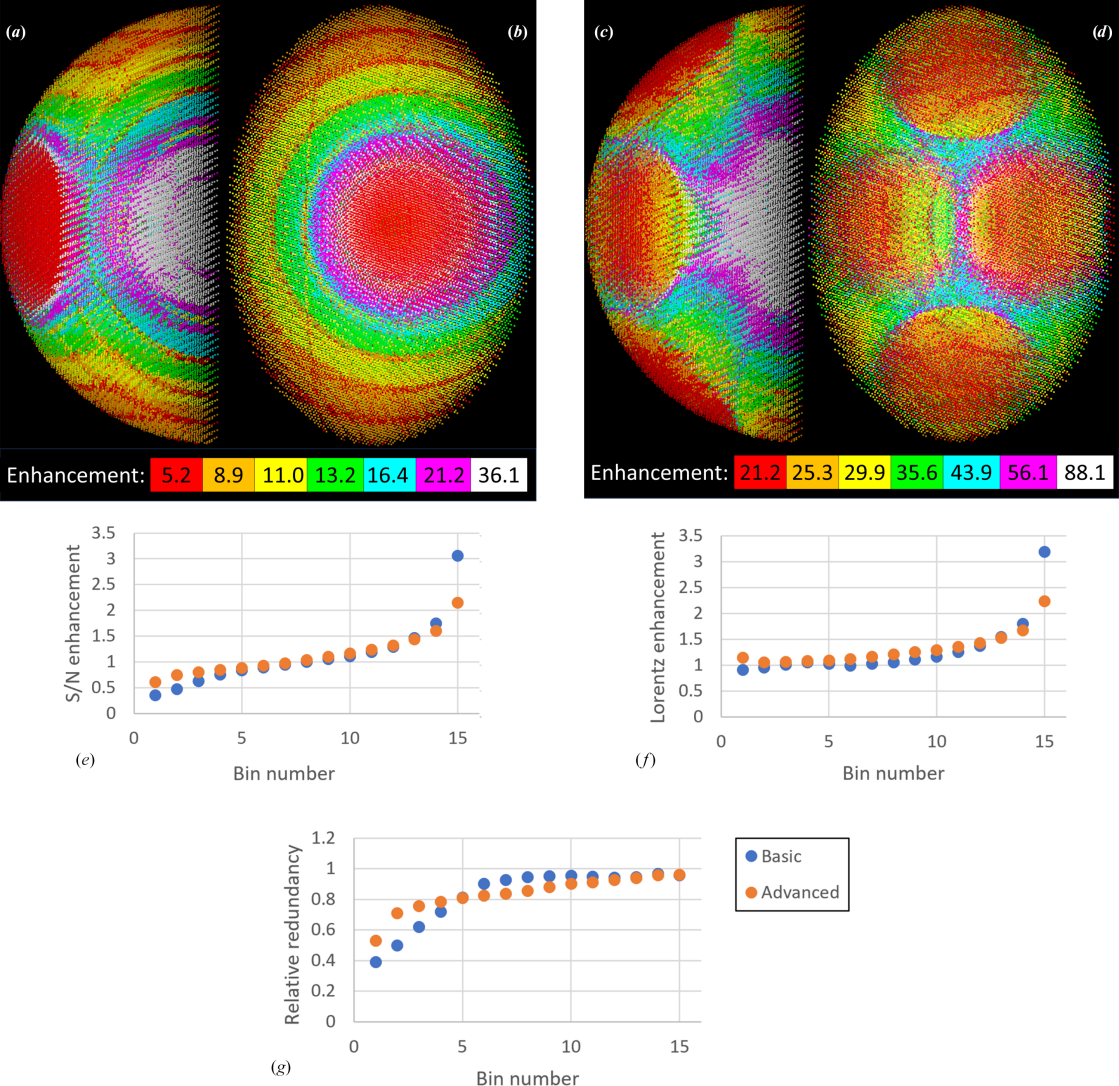
Merged reflection S/N enhancement factors in equi-populated bins for the experiment described in Fig. 9 ▸. (*a*, *b*) Two views of the enhancement distribution for the basic (single-sweep) experiment. Note that the apparently simple image in (*a*) arises from an angled plane cutting across a complex three-dimensional pattern; see Fig. 5 ▸ for comparison. (*c*, *d*) Two views of the enhancement bins for the advanced (four-sweep) experiment. Note the more even spread of red and orange points in this experiment, and the narrower spread between the highest and lowest enhancement factors. (*e*, *f*, *g*) Distribution of S/N enhancement factors in the merged reflection file for the basic and advanced strategy, corrected for the effect of resolution. (*e*) shows the S/N enhancements, (*f*) the Lorentz-dependent contribution and (*g*) the redundancy-dependent contribution. The reflections have been sorted and binned by S/N enhancement, and have been adjusted to an equivalent redundancy of 1 for both basic and advanced strategies.

Fig. 10 ▸ shows the S/N enhancements in the merged reflection file for basic (single-sweep) and advanced (four-sweep) strategies. The enhancement is dominated by the resolution of the reflections, and the contribution to non-uniformity from redundancy and Lorentz factor, respectively, is seen to be comparable. It is clear from the figure that the advanced strategy gives a significant enhancement increase to the less enhanced reflections, by averaging the cusps, the image of the module gaps and the high Lorentz factor regions more evenly across the reflections.

The best practical approach seems to be to combine a number of different orientations and their symmetry images so as to spread them uniformly across reciprocal space while avoiding overlap between cusps. The best way to do this is determined by the crystal symmetry and by which orientations are accessible. The description below shows the preferred combinations of orientations, although for certain crystal orientations, notably when the φ axis is close to a crystal symmetry axis, these preferred orientations cannot in practice be achieved and alternative sets of orientations must be used; for a goniostat with a maximum χ of 48° it is only possible to reach 33% of the possible crystal orientations.

**Figure 11:**
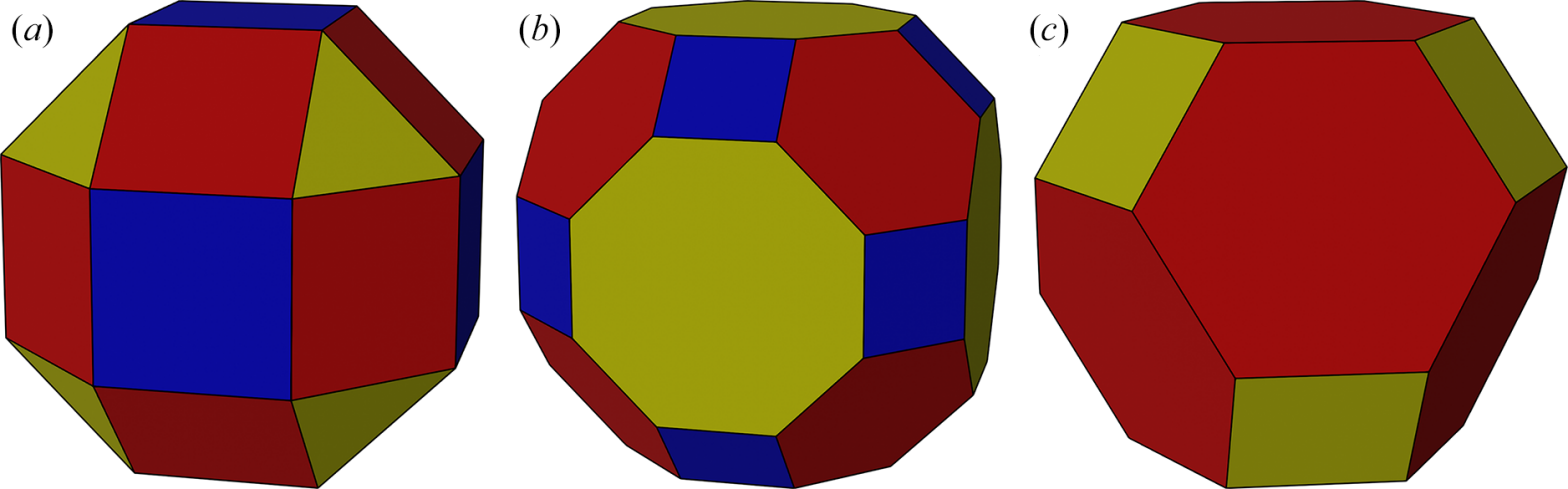
Rhombicuboctahedron (*a*), truncated cuboctahedron (*b*) and truncated octahedron (*c*).

The aim of the strategies is to spread the measuring orientation and associated cusps (and their symmetry equivalents) evenly across reciprocal space. The vertices of regular polyhedra fulfil this requirement, providing sets of points that are uniformly spread across the sphere and so give a good basis for selecting orientations. When the crystal point-group symmetry is a subset of the polyhedron symmetry, it is possibly to generate the appropriate distribution of symmetry images with only a few orientations. The orientation vectors are positioned so that they match selected vertices of the polyhedron, and the symmetry operations generate a result equivalent to having an orientation vector at every pair of vertices. The rhombicuboctahedron (Fig. 11 ▸*a*) with 24 vertices has been the basis of a number of strategies. In point group 23 it is sufficient with a single orientation that matches any vertex, while point groups 222 and 4 require a triplet of orientations that match one of the triangles in the figure. In point group 3 the requirement is for four orientations that match the square of points sitting between four different triangles (although an alternative strategy using only three sweeps is also used). These sweep combinations will be achievable over a large fraction of the possible crystal orientations. The truncated cuboctahedron (Fig. 11 ▸*b*) is the basis of a single-sweep strategy for point group 432 and a two-sweep strategy for point group 23, while the truncated octahedron (Fig. 11 ▸*c*) is the basis of a two-sweep strategy for point group 32. For point groups 1 and 2 (and, for some orientations, 3) the range of orientations provided by the goniostat is not wide enough to give a full coverage of the sphere.

**Figure 12:**
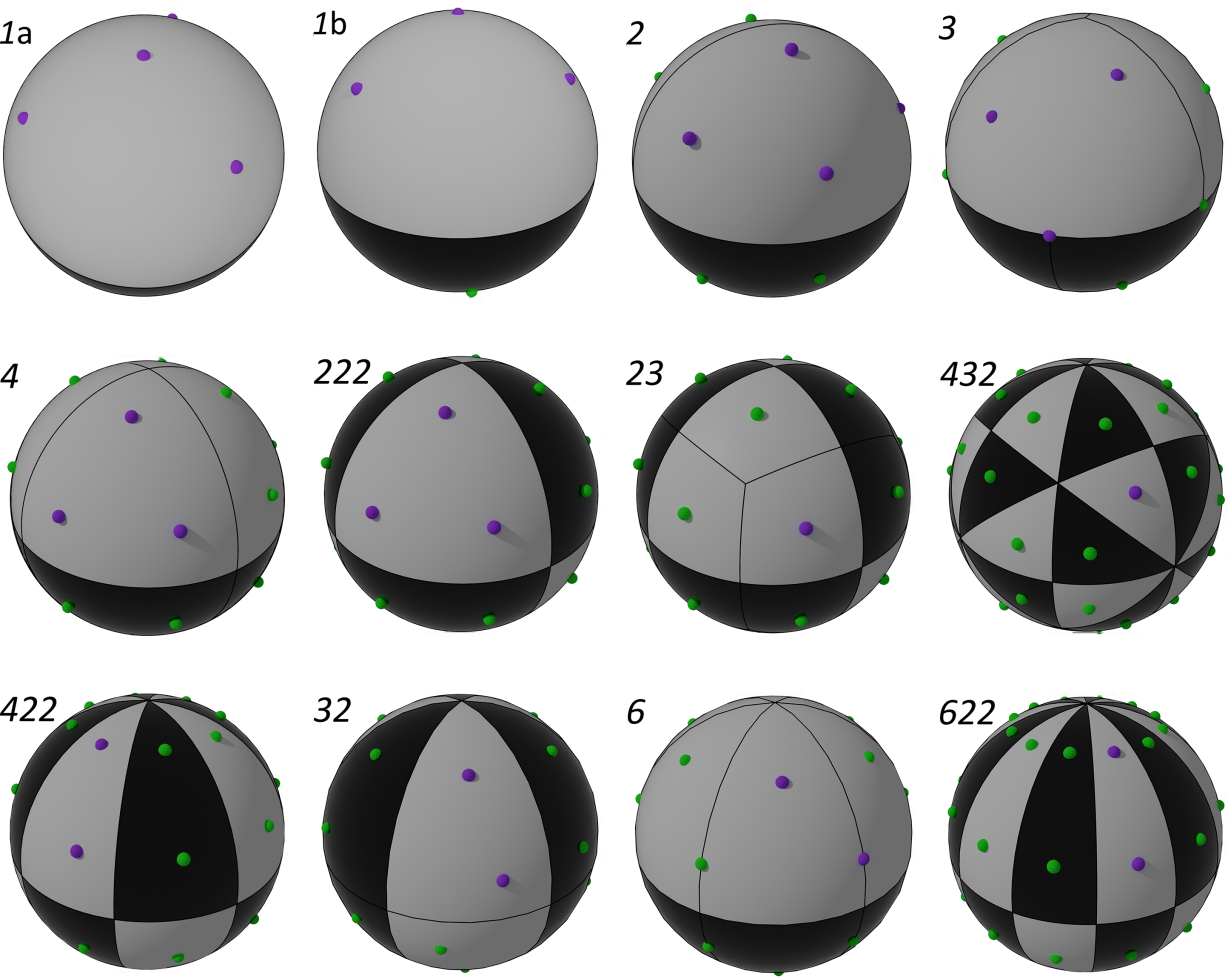
Target orientations for advanced strategies for the 11 point groups shown in the orthonormalized reciprocal crystal coordinate system. The actual orientations of sweeps are shown in purple, while their symmetry images are in green. Each tile contains a full set of unique (non-anomalous) reflections; a complete set of anomalous reflections requires the combination of a white and a black tile. Strategies for 4, 222 and 23 correspond to a rhombicuboctahedron (Fig. 11 ▸*a*), the strategy for 432 to a truncated cuboctahedron (Fig. 11 ▸*b*) and the strategy for 32 to a truncated octahedron (Fig. 11 ▸*c*). The *P*1 strategy is depicted from two different angles (1a and 1b) in order to show all four orientations. The strategy shown for 3 is the three-sweep version.

For point groups 6, 422 and 622 multiplicities are already high, and it is not possible to achieve a spherically symmetric distribution, so we simply aim for a two-sweep strategy. The target strategies for the different point groups are shown in Fig. 12 ▸.

The sweep requiring the lowest value of χ is acquired first and is set to a length of 360°, as this gives the best basis for scaling calculations. Successive sweeps may be acquired with 360° as well, if this is possible without goniostat shadowing, but are otherwise set at 192°. The number of sweeps, experiment length and resulting multiplicity for the different strategies is shown in Table 2 ▸.

**Figure 13:**
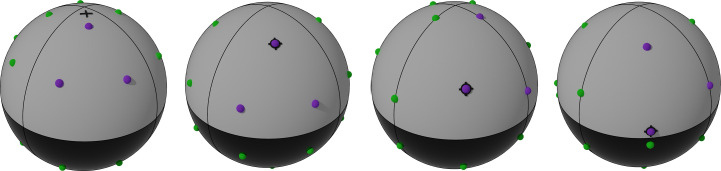
Strategies used for tetragonal crystals shown in the orthonormalized reciprocal crystal coordinate system. The actual orientations of sweeps are shown in purple and their symmetry images in green. The orientation of the φ axis shown as a black cross. The strategies have been changed from the ideal polyhedron-based strategies in order to run the first sweep at χ = 0°, reducing the number of crystal-recentring operations required by one.

Even after all these considerations there are still trade-offs to be made, notably whether to try to acquire the first sweep at χ = 0°, shortening the experiment by one centring at the cost of a less ideal distribution of orientations. Fig. 13 ▸ shows an example of the strategies used for tetragonal crystals when starting with a sweep at χ = 0°. These choices will ultimately depend on the operational timings at different synchrotrons and the priorities of the users; the workflow may offer either one or the other, or a choice of both, depending on circumstances.

### 4.6. Phasing strategies

Phasing experiments rely on measuring small intensity differences between reflections that in the absence of anomalous scattering should have the same intensity at a given wavelength. The single-wavelength anomalous dispersion (SAD) method is based on comparing the intensities of reflections *h*, *k*, *l* and −*h*, −*k*, −*l* (or its symmetry mate). The multi-wavelength anomalous dispersion (MAD) method, besides using the same comparisons as the SAD method at each wavelength, compares reflections measured at different wavelengths close to an absorption edge. In either case, the overriding concern is that the reflections being compared should be measured so that the irradiated volume, beam intensity, radiation damage and generally scaling be as similar as possible to avoid compromising the intensity difference measurements. For SAD it is possible to ensure that the pairs of reflections to be compared appear on the very same image by aligning the crystal along an evenfold symmetry axis. If we assume that the axis in question is along *Z*, the two reflections *h*, *k*, *l* and *h*, *k*, −*l* will appear on the same image. Since the symmetry axis ensures that the intensity of *h*, *k*, −*l* is the same as that of −*h*, −*k*, −*l*, the two intensities to be compared will have exactly the same scaling. If the alignment of a symmetry axis is not possible, the best alternative is ‘interleaving’, *i.e.* measuring a small ‘wedge’ of images at ω then at ω + 180°, so that reflections *h*, *k*, *l* and −*h*, −*k*, −*l* are measured from the same irradiated volume and with similar degrees of radiation damage. Similarly, data for MAD phasing can be acquired with wavelength interleaving. An alternative approach is to measure with high multiplicity and make sure that each complete data set is acquired in the shortest possible time, so as to at least reduce the spread of radiation damage within each block of the data set.

**Figure 14:**
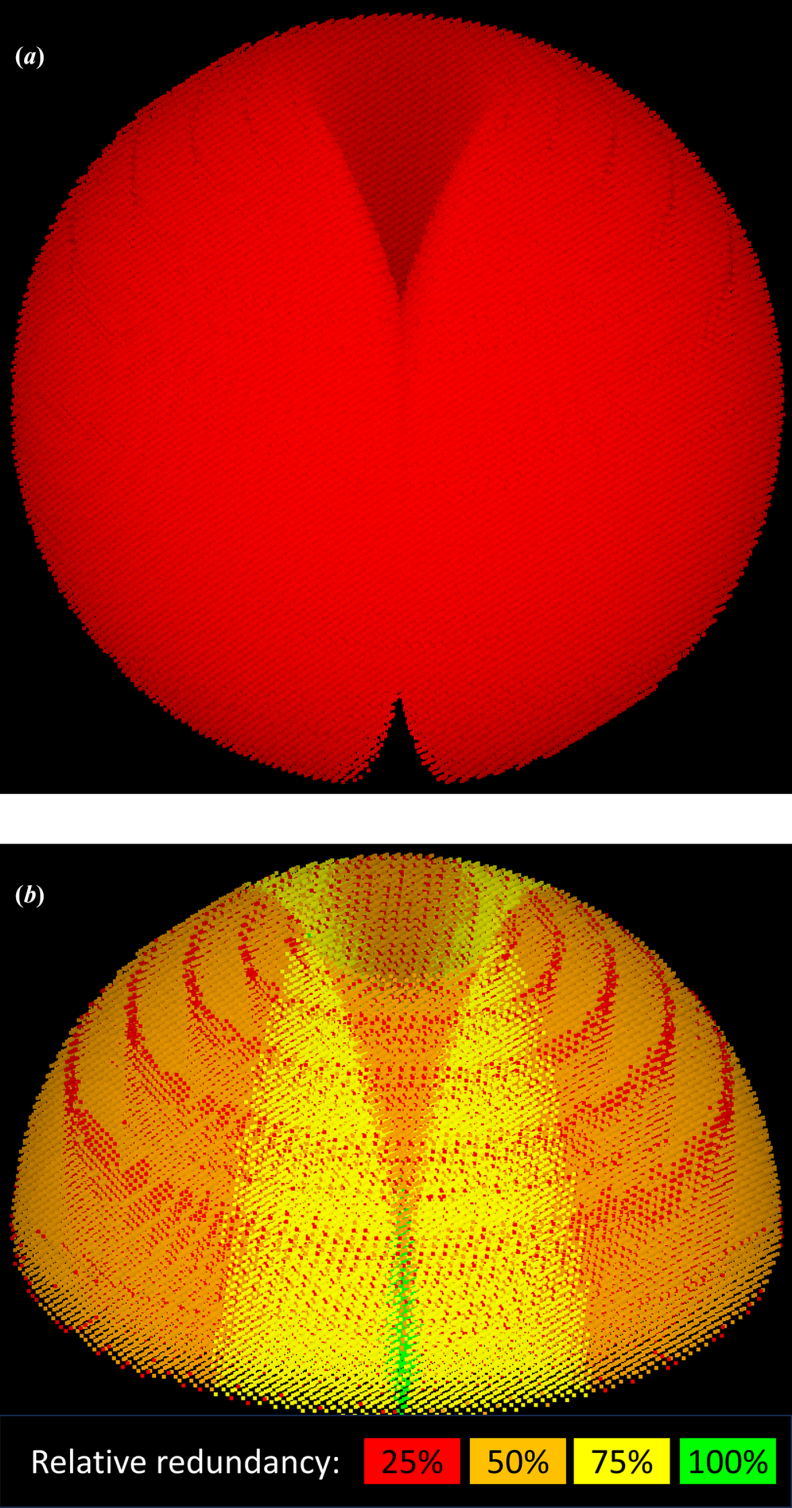
Raw reflections (*a*) and merged reflections (*b*) as measured by a sweep of length 180° + 2θ_max_ for a triclinic crystal. Simulation parameters are as for Fig. 4 ▸. (*b*) shows the merged reflections for one hemisphere, colour-coded by non-anomalous redundancy relative to a 360° sweep. The orange triangle between the two yellow wedges are points where only half of the anomalous pair has been measured; red points arise from reflections lost in the detector-module gaps.

The minimum sweep length needed to achieve close to 100% anomalous completeness in *P*1 is 180° + 2θ_max_ (see Fig. 14 ▸), which still excludes a volume somewhat larger than the cusps.

The work of Dauter (1999 ▸) gives the shortest sweeps that can give complete data sets for a number of different orientations. An approximate summary would be: in *P*1, 180° + 2θ_max_; in single-axis point groups, 360°/*n* aligned on an *n*-fold axis or 90° + θ_max_ aligned orthogonal to a symmetry axis; in other point groups, 360°/2*n* aligned on an *n*-fold axis (*n* even) or 90° aligned orthogonal to a symmetry axis. [It should be noted that Dauter (1999 ▸) gives the minimum sweep length for anomalous completeness orthogonal to the twofold axis in a monoclinic crystal as 180° + 2θ_max_; to the present authors this appears to be incorrect.] To obtain full completeness it is frequently necessary to start the sweep at particular values of ω that match the direction of crystal symmetry axes. This may conflict with the ω values that would minimize shadowing. For sweep lengths of the order of 180° it may be possible to obtain the desired effect by dividing a sweep into two parts and acquiring them out of sequence, so that the first and second half of the acquired images each gives 100% completeness.

**Figure 15:**
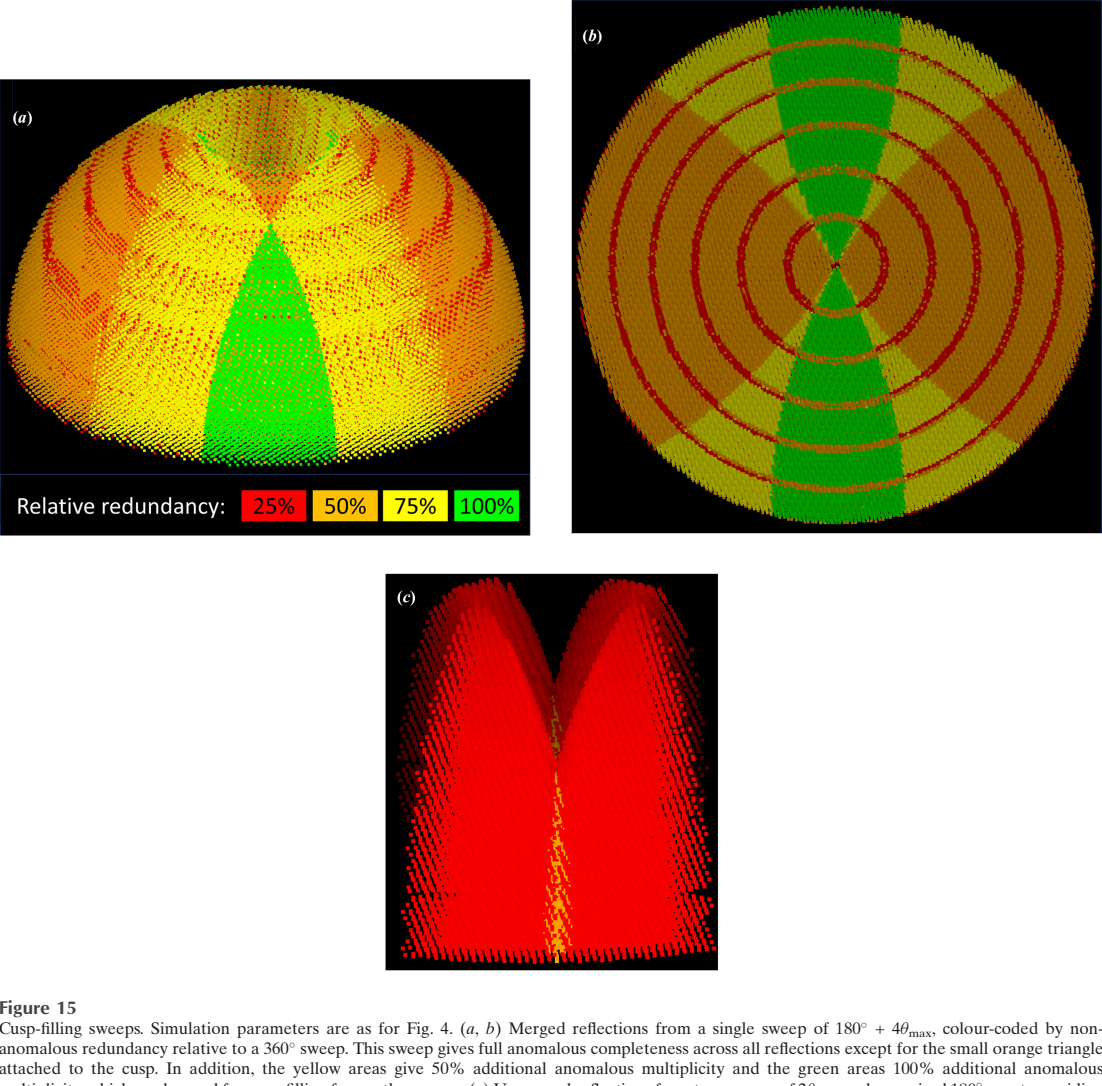
Cusp-filling sweeps. Simulation parameters are as for Fig. 4. (*a*, *b*) Merged reflections from a single sweep of 180° + 4θ_max_, colour-coded by non-anomalous redundancy relative to a 360° sweep. This sweep gives full anomalous completeness across all reflections except for the small orange triangle attached to the cusp. In addition, the yellow areas give 50% additional anomalous multiplicity and the green areas 100% additional anomalous multiplicity, which can be used for cusp-filling for another sweep. (*c*) Unmerged reflections from two sweeps of 2θ_max_ each, acquired 180° apart, providing 100% anomalous multiplicity to fill the cusp from another sweep.

An additional problem in phasing experiments is that it is frequently necessary to ‘fill in’ cusps, particularly when aligning on symmetry axes where the resulting cusp is completely uncompensated. The ideal cusp-filling sweep should be at an orientation as far as possible away from the cusp and of a length of 180° + 2θ_max_ (for basic completeness) or more (for additional filling-in of the cusp). Fig. 15 ▸ shows some approaches to this problem, but because of difficulties with goniostat shadows, additional filling-in would tend to be impossible at higher resolution.

The best phasing strategies vary significantly depending in particular on which axis alignments are possible at a given crystal orientation, and it is not fruitful to refer to a single ‘preferred’ strategy for each point group. In practice one has to combine the available building blocks in a number of different ways, depending on the precise orientation of the crystal and the desired number of orientations.

## 5. Workflow implementation

**Figure 16:**
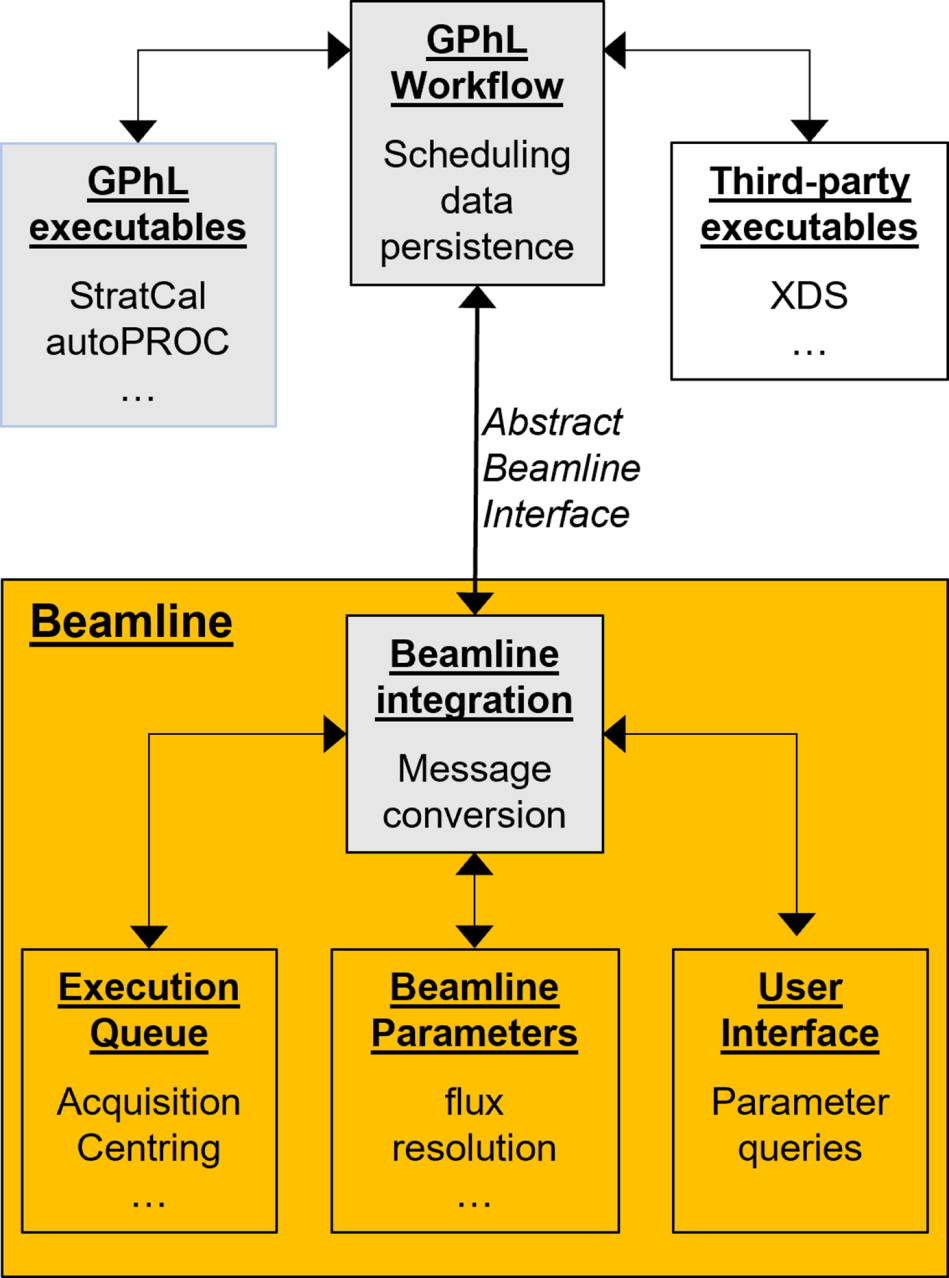
Implementation and integration of the *GPhL* workflow. *Global Phasing* software is shown in grey and beamline-control system software in orange.

The implementation and integration of the *GPhL* workflow is shown in Fig. 16 ▸. The workflow development began in a collaboration with the Diamond Light Source for use at its I23 beamline. I23 is a specialized beamline designed originally for low-energy phasing experiments, and features a full-κ goniostat, *in vacuo* operation and a ‘semi-cylindrical’ PILATUS 12M detector with a 2θ range of 200°, which presents a number of particular problems. The focus of the work later shifted to dealing with more ‘normal’ detectors and integration with *MXCuBE*, due to the increased availability of developer resources and more open software architecture provided. From the beginning the workflow was designed as a beamline-agnostic, standalone program that could be interfaced with different instruments and beamline-control systems. At present the workflow is available as integrated with the *MXCuBE* beamline-control system (Gabadinho *et al.*, 2010 ▸; Oscarsson *et al.*, 2019 ▸) which, being an open-source collaboration, allowed much of the integration to be performed by *GPhL*. Integration with other systems is possible given the access and synchrotron resource commitments necessary for the rewriting of the beamline integration module.

The workflow engine (written in Java) runs in a separate process and communicates with the beamline-control system by message passing (currently using the Py4J library for communication to Python beamline code). Messages are Java objects matching an Abstract Beamline Interface (Keller, 2012 ▸, 2014 ▸) that is designed to structure the required information in a beamline-independent manner. On the beamline side there is an adaptation layer that translates the messages from Java, queries and sets parameters, and interacts with the native user interface and acquisition queue. The workflow engine takes care of data persistence, and dispatches jobs to external programs, such as *XDS* (Kabsch, 2010 ▸), *StratCal*or *autoPROC* (Vonrhein *et al.*, 2011 ▸). The final task of the workflow is to trigger an *autoPROC* processing job, using a bespoke wrapper (aP_wf_process) to transfer complete information about the relationship between different sweeps, indexing settings, expected symmetry *etc*. to the processing program.

## 6. Availability

At the time of publication, the *GPhL* workflow is available in integration with *MXCuBE* and has been in production use at DESY P14 in Hamburg for five years, acquiring 500–1000 data set per year on over 15 different projects. The workflow has been run successfully at the ESRF ID30B and MASSIF-1 beamlines, ALBA XALOC, SOLEIL PX2 and MAX IV BioMAX, and collaborations are under way with a view to deploying it for production. Further collaborations are being explored. The *GPhL* workflow can be made available to other beamlines who are willing to collaboratively implement and deploy the Abstract Beamline Interface under their beamline-control system.

## 7. Conclusions

The benefits of the strategies presented here are hard to quantify exactly, because of the need to balance so many competing factors that each needs to be evaluated separately. One clear benefit is that the workflow-driven strategies allow you to run quite complex experiments either automatically or with minimum input, which would otherwise have been impractical. Many of the features in the workflow are designed to improve the worst-case outcomes: the handling of crystal orientations avoids the multiplicity reduction arising from cusps and module gaps, the standardized parameter values and the automatic transmission setting reduces unfortunate or ill-informed user choices, and the multi-image characterization and the attention to scaling and goniostat shadow compensation makes for more robust processing. It may be that the clearest advantage comes from the systematic use of high-multiplicity–low-transmission multi-sweep experiments, which would be a lot more complex to set up manually. The best practical example of the advantages of the *GPhL* workflow and strategies is probably that shown in Table 3 ▸ taken from Donath *et al.* (2023 ▸); see also Schulze-Briese *et al.* (2022 ▸).

## Figures and Tables

**Figure 7:**
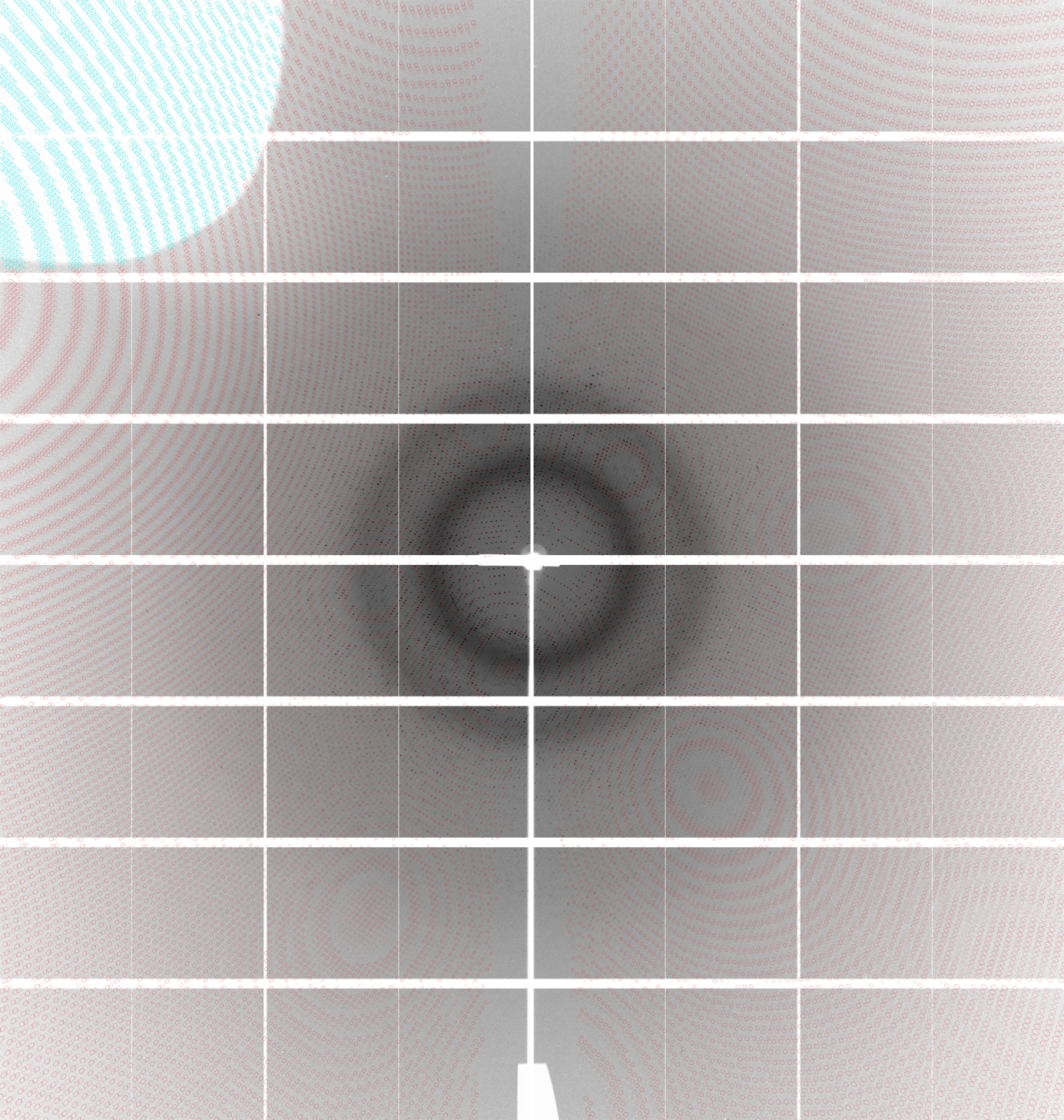
Detector image, showing the goniostat shadow (in white) and reflections predicted by *autoPROC* to be visible (red) or shadowed (light blue). The image was measured at the P14 beamline at the DESY synchrotron in Hamburg under production conditions.

**Table 1:** χ_max_ and best resolution for representative detector types 2θ_max_ was calculated from the narrowest detector dimension assuming a centred detector, a shadow cone half-angle of 19.84° (Arinax, personal communication) and a shortest detector distance of 137 mm (Gleb Bourenkov, personal communication).

Detector	Width (mm)	2θ_max_ (°)	χ_max_† (°)	Resolution‡ (Å)
EIGER 16M	311.1	48.6	21.6	1.22
EIGER 9M	233.1	40.4	29.8	1.45
PILATUS4 4M	311.1	48.6	21.6	1.22
PILATUS4 2M	233.1	40.4	29.8	1.45
PILATUS3 6M	423.6	57.1	13.1	1.04

† The highest χ value that allows a 360° sweep to be acquired without shadowing on the detector.

‡ The highest resolution that can be made to fit on the detector for 12.398 keV radiation.

**Table 2:** Current version of advanced native strategies Strategy lengths are given as the sum of one 360° sweep and a number of 192° sweeps. The multiplicity is the maximum multiplicity corresponding to the given strategy length, disregarding additional multiplicity arising from prolonging the sweeps from 180° to 192°.

Point group	Sweeps	Length (°)	Multiplicity
1	4	936	10
2	4	936	20
3†	3	744	24
3‡	4	936	30
222	3	744	32
4	3	744	32
32	2	552	36
6	2	552	36
422	2	552	48
622	2	552	72
23	1	360	48
432	1	360	96

† Three-sweep version.

‡ Four-sweep version.

**Table 3:** Quality factors for experiments on two identical orthorhombic crystals of a 560 kDa protein, each with a total dose of 1.5 MGy Note that the strategies used belonged to an earlier version of *StratCal*. Diffraction limits were determined using *STARANISO* (Tickle *et al.*, 2016 ▸).

	Std: 1 × 360°	*GPhL*: 1 × 360° + 2 × 210°
Diffraction limits (Å)	1.14, 1.07, 1.22	1.08, 1.09, 1.05
*R*_merge_/*R*_p.i.m._	0.132/0.037	0.123/0.027
Total reflections	25980668	49847294
Unique reflections	1892149	2320166
Completeness (%)	95.6	97.6
Mean *I*/σ(*I*)	12.8	16.1
Multiplicity	13.7	21.5
